# Effects of an educational program using a virtual social network on nurses’ knowledge and performance of blood pressure measurement: a randomized controlled trial

**DOI:** 10.1186/s12912-022-01137-0

**Published:** 2022-12-21

**Authors:** Mohamed E. H. Elzeky, Noha F. M. Shahine

**Affiliations:** 1 Medical-Surgical Nursing Department, Faculty of Nursing, Mansoura University, Dakahlia, Egypt; 2 Gerontological Nursing Department, Faculty of Nursing, Mansoura University, Dakahlia, Egypt

**Keywords:** Blood pressure measurement, Nurse, Distance learning, Performance, Knowledge

## Abstract

**Background:**

Hypertension diagnosis, treatment, and follow-up depend on accurate blood pressure measurement; however, inaccurate measurement techniques are common among healthcare providers. To improve professional performance, continuous education is necessary. Distance education through virtual social network can be used as it is easy to use and accessible.

**Methods:**

This study adopted a randomized controlled trial design and was conducted at two hospitals in Mansoura, Egypt. The subjects were selected from two hospitals using Stratified random sampling method in proportion to the total number of nurses. Seventy nurses were included in this study and were randomly divided into the intervention (*n* = 35) and control (*n* = 35) groups using block randomization. Data were gathered before and after intervention using a blood pressure measurement knowledge questionnaire and a blood pressure measurement observation checklist. The blood pressure measurement error (the difference between the BP determined by each observer and the reference BP) was calculated for the randomly selected recordings in both groups. The intervention group received 24 blood pressure measurement education sessions using WhatsApp, while the control group received only routine education using traditional lecture.

**Results:**

No statistically significant differences in pretest knowledge scores, performance scores, and range of error were found between both groups, whereas, after intervention, knowledge scores were higher in the intervention group than those in the control group (*p* < 0.001). The range of error of systolic and diastolic blood pressure values significantly reduced after the intervention in the intervention group, and the posttest performance scores were higher than the pretest performance scores; however, the difference was not statistically significant.

**Conclusions:**

The blood pressure measurement education program via WhatsApp was effective in increasing nurses’ knowledge and reducing the range of error; however, a multimodal approach may be required to improve performance scores.

**Trial registration:**

Prospectively registered with ClinicalTrials.gov on 09/03/2021; registration number NCT04789642**.**

**Supplementary Information:**

The online version contains supplementary material available at 10.1186/s12912-022-01137-0.

## Introduction

Hypertension (HTN) is a serious health problem affecting 1.3 billion individuals and accounts for 8.5 million deaths yearly worldwide [1]. Up to 75% of those deaths occur in low- and middle-income countries [2], such as Egypt, where the prevalence of HTN among adults reached 23% [3].

HTN diagnosis, treatment, and follow-up depend on accurate blood pressure measurement (BPM). Current guidelines discuss the fundamental principles of BPM and recommend the use of an automated BPM device to reduce observer errors [4–6]. However, in Egypt, healthcare practitioners, including nurses, use the auscultatory method to assess BP [7].

The use of an inaccurate measurement technique is common [8], and poor knowledge and practices regarding BPM are present among doctors and nurses in Africa [9]. Erroneous BPM can result in misdiagnosis and unnecessary or insufficient drug therapy, leading to avoidable and unnecessary burden of cardiovascular disease [5]. This renewed the concentration on BP educational programs, including basic training and continuing professional education [10].

Structured continuous training in accurate techniques is effective in enhancing clinical knowledge and performance of staff; however, the programs used in previous studies were held face-to-face for several hours, which consumed teaching and staff time and required high development cost [11–13]. Virtual social networks (VSNs) are inexpensive and are readily available so that they can be used as an educational instrument for nurses. WhatsApp is a widely used social networking smartphone application that can be integrated into teaching because of it is popularity and users do not need usage training [14, 15]. Studies have reported that distance learning and in person education have similar effects on nurses’ clinical skills [16]. Additionally, distance education provided more stable information and saved time and energy [17].

Thus, BPM can be performed with accurate results only through repeated comprehensive training programs [18]. In the clinical setting, nurses take the responsibility of BPM and assessment; however, studies conducted in Egypt have focused only on physicians, and no published studies have examined nurses regarding this knowledge and practices in BPM [19]. Globally, nurses face great shortages in personnel, leading to heavy work load and less time for education and development [15, 20, 21]. Only studies on face-to-face training have reported significant improvements in BPM skills and knowledge [22, 23], whereas a study on web-based training has revealed insignificant improvement in BPM skills and knowledge [13]. Finding new methods for distance learning is a major concern. Distance learning through VSNs does not require the participants to attend the sessions in person and offers unlimited access to the intervention materials whenever the participants want [24]. Additionally, no published study has investigated BPM education using VSNs.

## Study aims

This study was designed to evaluate the effects of an educational program using WhatsApp on nurses’ knowledge, performance, and error range of BPM. It was hypothesized that nurses who had received educational program via whatsapp would have improved BPM knowledge, performance and accuracy of readings than nurses who had not enrolled in the whatsapp educational program.

## Methods

### Study design

This study was a parallel, single-blind, randomized controlled trial with a pretest, posttest, and control group.

## Subjects

**Fig. 1:**
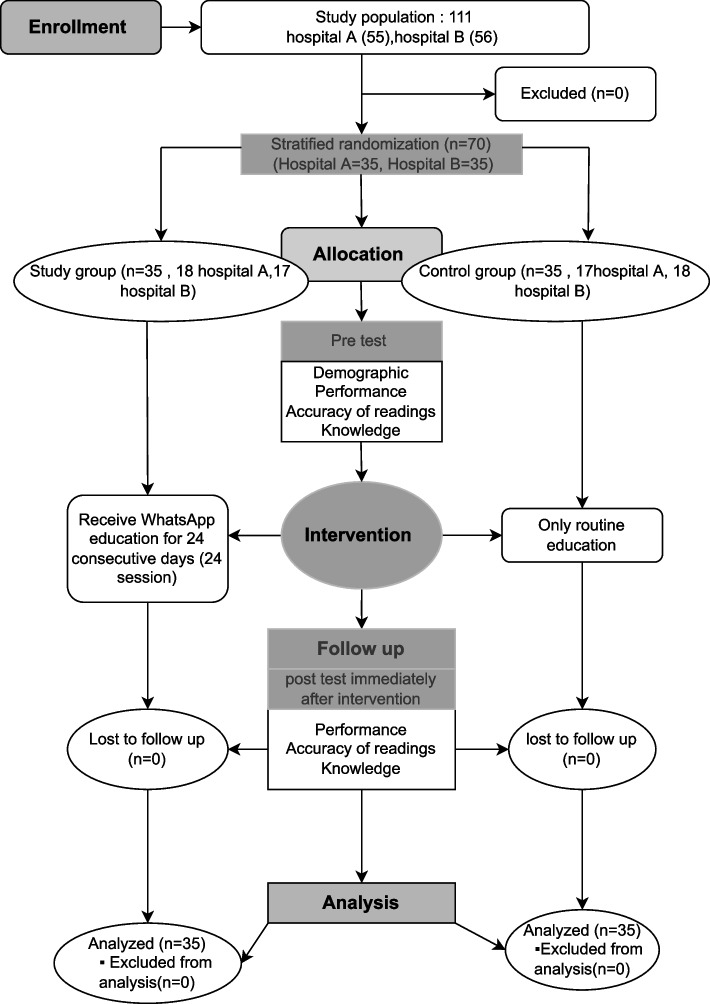
Study flow diagram

This study was conducted in two cardiac departments of the specialized medical hospital and the main university hospital affiliated to Mansoura University from the period of 15^th^ march to 15^th^ may 2021. The subjects were selected using Stratified random sampling method in proportion to the total number of nurses. The two hospitals were considered as strata with a total of 55 nurse working in Hospital A (medical specialty) and 56 working in hospital B (surgical specialty). Based on data from the study by Machado et al., [23] considering the level of significance of 5% and power of study of 80%, with a two-tailed study design, the sample size was calculated using the following formula: *n* = [(Zα/2 + Zβ)2 × {2(SD)2}]/(difference)2, where SD indicates standard deviation, the value of Zα/2 depends on 5% significance level (1.96), and the value of Zβ depends on 80% power (0.84). Therefore, *n* = [(1.96 + 0.84)2 × {2(2)2}]/(1.35)2 = 34.4. Based on the aforementioned formula, 35 nurses were randomly selected using Microsoft Excel RAND function from each hospital. The study population consisted of 70 nurses and they were randomly assigned to the intervention and control groups, using block randomization with a block size of 4. All randomization procedures were performed by independent statistician and were blinded to authors until intervention procedures. The nurses were recruited using the inclusion criteria that included agreement to participate, constant staff worked at the mentioned setting, both sexes, and the ability to install WhatsApp in their smartphones. The exclusion criteria were as follows: nurses with hearing or visual problems, and those who failed to complete the pretest tools (Fig. 1).

### Tools

The two tools used in this study included the Blood Pressure Measurement Knowledge Questionnaire, and the Blood Pressure Measurement Observation Checklist.

### Tool I. Blood Pressure Measurement Knowledge Questionnaire

This tool was adapted from the study by Du Toit [25] to assess theoretical knowledge and was updated and modified by the researchers according to the American Heart Association (AHA) 2019 guidelines for BPM in humans [5] and International Society of Hypertension (ISH) guidelines [6]. It involved five sections. Section 1 concentrated on demographic data, including age, gender, experience, educational level, hospital, department, and last BP training. Sections 2–5 focused on background knowledge (6 items), patient preparation (8 items), patient positioning and equipment (9 items), proper measurement technique (10 items), and hypertension diagnosis and documentation (8 items). The scoring system was applied by adding 1 point and 0 point to each correct and incorrect answer, respectively. Then, the total and the percentage mean scores were calculated. The knowledge level was poor, fair, and good if the percentage scores were less than 50%, from 50% to < 75%, and more than 75%, respectively [19].

### Tool II. Blood pressure measurement observation checklist

This checklist was adapted from the study by Du Toit [25] for practical assessment in which the subjects demonstrated their practical technique knowledge. It was updated and modified by the researchers according to the AHA 2019 guidelines for BPM in humans [5] and ISH guidelines [6]. It involved five parts. Part 1 focused on the subjects’ ability to identify the Korotkoff sounds correctly. Parts 2–5 focused on measurement steps related to patient preparation (8 items), patient position and equipment (7 items), proper measurement technique (9 items), and hypertension diagnosis and documentation (4 items). Five videos were randomly selected (i.e., BP 2, BP 4, BP 7, BP 23, and BP 24) from the British and Irish Hypertension Society (BIHS) training database [26]. The database covers several clinical situations, involving healthy individuals’ recordings, patients with different arrhythmia types, and common conditions in daily clinical work. The reference answers of the readings were also obtained from the BIHS and were validated by 24 experts. Each nurse was blinded to the reference answers. This database was previously used as an assessment tool by Zhang et al.[27] a written permission was obtained from BIHS to use database in our study. Randomly selected Videos were the same for all nurses at pretest or posttest, and the mean overall score was calculated and compared with that in the reference answer. The acceptable mean range of error (ROE) was within (-2 to + 2) mmhg [11]. A scoring system was applied by adding 1 point and 0 point to correct and wrong steps, respectively. Then, a total score was estimated for performance items, and the mean percentage score was calculated. The level of performance was considered poor, fair, and good if the scores were less than 60%, from 60 to 80%, and more than 80%, respectively [19].

## Data collection

This is a single blinded trial where both data collectors and students (mock patients) were blinded to study groups during the entire study period. Data were collected by research assistants who first, performed the practical assessment while the participant nurses consulting a standardized patient. Internship nursing students acted as standardized patients, who were normotensive students with a medium upper arm circumference (not exceeding 34 cm). They were trained in posture, behavior, responses, and standardized attitudes to be replicated. So, they systematically reproduced identical history for the consultations and acted the same way in all consultations. The measurement technique was assessed using tool II, and the participants’ ability to identify the Korotkoff sounds correctly was assessed using five video clips from the BIHS (2017) [26] database. The video clips were played using Windows Media Player using the same computer and earphone in a quiet room, and the nurses recorded systolic and diastolic BP readings in a sheet. After demonstrating the technique, demographic characteristics and knowledge level were collected using tool I. All questionnaires take about (60–90) minutes to be completed for each participant.

## Intervention

A social group was created in WhatsApp for the subjects in the intervention group, and they received 24 consecutive educational sessions about BPM guidelines once daily as video clips, texts, images, audio clips, and video presentations by the first author, in addition to the routine BPM education provided by the hospital staff development team. Each education session take 5 to 7 min to view, in addition to open discussion that last all over the day. The number of education sessions was determined depending on the BIHS BPM auscultatory tutorials. It includes 24 eligible videos of Korotkoff sound recordings (after exclusion of the five videos used in the pretest and posttest). The educational content involved materials presented in the AHA guidelines for BPM [5] and ISH guidelines [6]. The education outlines were importance of accurate BPM, common BPM errors, types of BP monitoring, proper Patient, equipment and environmental preparations, cuff size and arm position, Cuff placement and stethoscope, proper BPM technique, Devices validation and recalibration, Body position and BPM, Kortokoff sounds, Terminal digit preference, appropriate measurements required for diagnosing and treating high BP or HTN, categories of BP among adults, accurate documentation and providing patient with BP readings and BPM considerations in Special Populations. The nurses’ online availability was evaluated daily to ensure that they read the materials. Moreover, questions were asked at the end of every session, and the nurses should send a “done” message via WhatsApp to the researcher. The nurses can send any questions regarding the study topic in the form of messages to the researcher via WhatsApp. The control group received only routine education provided by the continuous hospital staff development team during the study and composed of 1 h traditional lecture training regarding BP measurement. Following the posttest phase, the educational materials were also provided to the control group.

### Validity and reliability

All tools were face-validated by a jury of five cardiac specialists in the field of medicine and nursing, and any necessary modifications were modified. Tool I had an average content validity index (CVI) of 0.93, while tool II had an average CVI of 1.0. A pilot study was conducted involving 20 cardiac nurses working at a private hospital to test the clarity and reliability of the tools. The nurses included in the pilot study were excluded from this study. The Kuder–Richardson reliability coefficient was 0.753 for tool I and 0.786 for tool II. The test–retest correlation was 0.84 for tool I and 0.910 for tool II.

### Ethical consideration

Ethical approval was obtained from the Research Ethics Committee, Faculty of Nursing, Mansoura University (research no. 0198). Official written permission to perform the study was obtained from hospital directors. Informed consent was obtained from all participants in the study.

## Statistical analysis

Data were analyzed using inferential statistics (i.e., paired t-test and independent t-test for normally distributed data; Wilcoxon test and Mann–Whitney test for non-normally distributed data; chi-square test and Fisher’s exact test for categorical data), and descriptive statistics (i.e., means ± standard deviations and frequencies). The Shapiro-Wilke test was used to evaluate the normal distribution of data. Data that show normal distribution were; total baseline knowledge scores for both groups and total post knowledge score in control group; total performance scores for both groups, and pre, post diastolic readings in both groups. All data analyses were performed using Statistical Package for the Social Sciences, version 20, and differences with *p-*values of less than 0.05 were considered statistically significant.

## Results

### Demographic characteristics

No significant differences in education level, gender, age, duration of BPM, the ability to read BPM guidelines, hearing and vision examination scores, and last BP training were found between both groups, indicating the homogeneity of the groups. (Table 1).

**Table 1:** Demographic characteristics of the study participants

**Variables**	**Study group (*n* = 35)**		**Control group (*n* = 35)**		**Significance**
	No	%	No	%	
**Sex**					
Male	25	71.4	27	77	χ^2^=0.299 *P* = 0.584
Female	10	28.6	8	22.9	
**Education level**					
Bachelor	18	51.4	22	62.8	χ^2^=1.271 *P* = 0.530
Diploma	10	28.6	9	25.7	
Post-graduate	7	20	4	11.4	
**Mean age**, year, mean ± SD	28.7 ± 3.8		27.9 ± 3.5		*Z* =-0.923 *P* = 0.356+
**Duration of BPM**, years					
<3	4	11.4	8	22.9	χ^2^ = 4.127 *P* = 0.248
3 to <7	8	22.9	4	11.4	
7 to <11	11	31.4	7	20	
≥11	12	34.3	16	45.7	
**Read BPM guidelines**					
No	25	71.4	23	65.7	χ^2^ = 0.265 *P* = 0.60
Yes	10	28.6	12	34.3	
**Last BP training**,** years**					
<1	5	4.3	3	8.6	FET=1.740 *P* = 0.645++
1 to <5	14	40	12	34.2	
5 to <10	5	14.3	4	11.4	
≥10	11	31.4	16	45.7	
**Hearing exam last year**					
No	25	71.4	22	62.8	χ^2^= 0.583 *P* = 0.445
Yes	10	28.6	13	37	
**Vision exam last year**					
No	15	42.8	11	31.4	χ^2^ = 0.979 *P* = 0.322
Yes	20	57	24	68.6	

+ Mann–Whitney test; +  + Fisher’s exact test; chi-square test

### Knowledge scores

The differences in the mean pretest knowledge scores between both groups were statistically insignificant (Table 2). However, the mean posttest total knowledge scores were significantly higher in the intervention group than those in the control group (*p* < 0.001).

**Table 2:** Comparing the mean knowledge and performance scores between and within the study and control groups

	**Study mean %**		**Control mean %**		***P***^**a**^	***P***^**b**^	***P***^**c**^	***P***^**d**^
**Knowledge domains**	Baseline	post	baseline	post				
Back ground knowledge	60.9 ± 21.7	95.2 ± 8.6	62.4 ± 23	64.2 ± 21	Z=-212 *P*=0.832	Z=-5.98 *p*=<0.001*	Z=-4.86 *p*=<0.001*	Z=-1.00 *p*=0.317
Preparation of the patient	63.1 ± 19	82.6 ± 14.2	59.7 ± 17	61.1 ± 19.7	Z=-727 *P*=0.467	Z=-4.51 *p*=<0.001*	Z=-4.52 *p*=<0.001*	Z=-1.15 *p*=0.251
Position of the patient & equipment	51.7± 15.2	78.6 ± 13.8	46.6 ± 16.2	48.8 ± 19.2	Z=-1.67 *P*=0.10	Z=-5.55 *P*=<0.001*	Z=-5.07 *p*=<0.001*	Z=-1.58 *p*=0.114
Proper technique	41.2 ± 11.7	78.3 ± 13	43.1 ± 13.5	45 ± 14.1	Z=-0.46 *p*=0.645	Z=-6.47 *p*=<0.001*	Z=-5.20 *p*=<0.001*	Z=-1.41 *p*=0.157
Hypertension diagnosis and documentation	69.6 ± 18.7	79.6 ± 21.5	67.8 ± 17.7	65.7 ± 20.1	Z=-0.60 *p*=0.550	Z=-2.77 *p*=0.006*	Z=-2.07 *p*=0.039*	Z=-1.06 *p*=0.291
Total knowledge score	55.8 ± 11.6	81.8 ± 11.7	54.3 ± 12.3	55.3 ± 14.2	t=0.522 df= 68, *p*=0.604^±^	Z=-5.96 *p*=<0.001*	Z=-5.17 *p*=<0.001*	t=-1.50 df= 34, *p*=0.143^±±^
**Performance domains**								
Preparation of the patient	57.1 ± 23.5	57.9 ± 21	54.3 ± 20	52.9 ± 19.6	Z=-0.44 *p*=0.659	Z=-1.05 *p*=0.293	Z=-0.47 *p*=0.637	Z=-0.94 *p*=0.346
Position of the patient & equipment	55.1 ± 18.4	58.5 ± 16.5	55.9 ± 15.2	56.4 ± 11.5	Z=-0.07 *p*=0.942	Z=-1.08 *p*=0.279	Z=-1.91 *P*=0.056	Z=-0.07= *p*=0.946
Proper technique	71.7 ± 19.9	73.7 ± 21	67.9 ± 20	69.2 ± 19.6	Z=-0.79 *p*=0.431	Z=-0.98 *p*=0.326	Z=-1.50 *p*=0.134	Z=-1.00 *p*=0.317
Hypertension diagnosis & documentation	50 ± 21	55 ± 20.8	47.9 ± 25.9	48.6 ± 18.1	Z=-0.36 *p*=0.719	Z=-1.70 *P*=0.09	Z=-2.11 *p*=0.035*	Z=-0.26 *p*=0.796
Total performance score	61.8 ± 15.6	64.08 ± 14	58.2 ± 14.1	58.8 ± 13.9	t=1.033 df= 68, p=0.305^±^	t=1.590 df= 68, *p*=0.1 16^±^	t=-1.703 df= 34, *p*=0.098^±±^	t=-0.947 df= 34, *p*=0.350^±±^

*p < 0.05 significant

^a^Mann-Whitney baseline study and control

^b^Mann-Whitney post study and control

^c^Wilcoxon study

^d^Wilcoxon control

^±^Student’s t test

^±±^paired t test

### Performance scores

No statistically significant differences in the mean pretest performance scores were observed between both groups (Table 2). The mean posttest performance scores were higher in the intervention group than those in the control group; however, the difference was not statistically significant.

### Accuracy of reading

At the pretest, both intervention and control groups showed a significant systolic (*p* = 0.021 and 0.04, respectively) and diastolic BPM differences (*p* = 0.039 and 0.038, respectively) from the reference answer without significant difference regarding systolic and diastolic range of error (ROE) between the two groups (*p* = 0.631 and 0.339, respectively). However, the mean posttest systolic and diastolic ROE was significantly lower in the intervention group than that in the control group (*p* < 0.0001 and *p* = 0.0001, respectively) without significant differences from the reference answer (*p* = 0.899 and 0.160, respectively) for the intervention group (Table 3).

**Table 3:** Comparing the ranges of error of blood pressure readings between and within the study and control groups

	**Ref answer**	**Study group**	**Sig from ref answer**^**+**^	**Control group**	**Sig from ref answer**^**+**^	**ROE study**	**ROE control**	**ROE sig**^**+**^	**Group **	**acceptable ROE rate**		**Sig **
										**Yes **	**No **	
**Systolic pre**	196.4 ± 14.3	187.6 ± 6.9	Z^a^=-2.31, *p* = 0.021*	188.1 ± 7	Z^a^= -2.1, *p* = 0.04*	−8.8 ± 6.9	−8.3 ± 7	Z^a^ = −0.480, *p* = 0.631	Study (35)	(1) 2.9%	(34) 97.1%	χ^2^=0.000 *P* = 1.00
									Control (35)	(1) 2.9%	(34) 97.1%	
**Systolic post**	196.4 ± 14.3	196.2 ± 5.4	Z^a^= −0.13, *p* = 0.899	187.5 ± 6.6	Z^a^ = −2.01, *p* = 0.04*	−0.20 ± 5.4	−8.9 ± 6.6	Z^a^ = -5.73, *p* < 0.0001*	Study (35)	(20) 57.1%	(15) 42.9%	χ^2^=18.7 *P* = <0.0001*
									Control (35)	(3) 8.5%	(32) 91.5%	
**Sig**^b^						Z = −4.9, *p *< 0.0001*	Z = −1. 29, *p *= 0.20					
**Diastolic pre**	106.8 ± 9.1	102 ± 3.8	t = −2.14, df = 38, *p* = 0.039*	102.8 ± 2.7	t = 2.15, df = 38, *p* = 0.038*	−4.8 ± 3.8	−4 ± 2.7	t = −0.963, df = 68, *p* = 0.339	Study (35)	(9) 25.7%	(26) 74.3%	χ^2^=0.324 *P* = 0.569
									Control (35)	(7) 20.0%	(28) 80.0%	
**Diastolic post**	106.8 ± 9.1	104.6 ± 1.19	t = −1.43, df = 38, *p* = 0.16	103 ± 2.5	t = 2.09, df = 38, *p *= 0.04*	−2.2 ± 1.19	3.8 ± 2.5	t = 3.49, df = 68, *p *= 0.001*	Study (35)	(17) 48.5%	(18) 51.5%	χ^2^=5.04 *P* = 0.025*
									Control (35)	(8) 22.8%	(27) 77.2%	
**Sig**^**++**^						t = 4.13, df = 34, *p* = 0.0002*	t = −0.394, df = 34, *p *= 0.696					

^*^p < 0.05 significant, ^**+**^ Student’s t-test., ^++^ Paired t-test., ^a^ Mann–Whitney test, ^b^ Wilcoxon test, chi-square test

## Discussion

Accurate BPM is important in the diagnosing and managing HTN. The reliability of BPM needs education, the recognition of recommendation implementation barriers, and engagement of the entire team [28]. Releasing staff from practice to present classroom teaching or arranging their release and fitting training according to their shifts is difficult. Thus, online learning could be useful through which staff can undertake their professional development [29]. We used WhatsApp as an educational tool in this study.

At baseline, the knowledge scores of both groups were at a moderate level, revealing inadequate hospital training on BPM for nurses. The findings of this study revealed that only approximately half of the participants in both groups received training on BPM in the last 5 years, suggesting a significant lack of institutions, and possibly nurses that can perform accurate BPM. This inadequate knowledge level has been reported in other studies as well. The studies by Machado et al., [23] and Du. [25], have reported a moderate level of all BPM domains. Another study by Machado et al., [30] has reported that cardiac nurses had poor theoretical knowledge on indirect BPM with only approximately half of the participants received training in the last 2 years after their professional education. Another study conducted at Nigeria has reported that most healthcare workers did not read guidelines and had poor BPM knowledge level [9].

After the intervention, the mean posttest knowledge scores in the intervention group significantly increased to high levels compared with pretest scores. Moreover, the mean posttest knowledge scores were significantly higher in the intervention group than those in the control group. Hence, education via VSNs increased nurses BPM knowledge. Few studies have examined the effect of VSNs on nurses’ education. These findings agree with those reported in a study involving nurses, which reported significant improvement in the knowledge level of nurses after telegram education [31]. Another study by Block et al., [13] has reported that knowledge on and practices of accurate BPM techniques improved significantly among nurses and medical assistants after a brief online continuous education program. In contrast, a study where nurses received a two-week intervention program has reported that social media posts did not significantly increase nurses’ knowledge on hypoglycemia management [32]; this may be due to some limitations, such as many posts per day and shorter intervention duration, causing fatigue and a decrease in the view rate.

This study showed that the mean pretest performance scores of the intervention and control groups were at moderate and poor levels, respectively. This inadequate performance level is found in other studies as well. A study by Machado et al., [23] has reported a moderate level of all BPM domains among nurses. Another study by Machado et al., [30] has reported that only 65% of BPM steps were followed by nurses. Another study by Manzoli et al., [33] has reported poor compliance to BP recommendations among healthcare workers. In contrast, a study involving physicians has reported good adherence to BP measurement guidelines [34]; this suggests that adherence evaluation was self-reported.

The findings of this study showed that following the intervention, no significant difference in BP performance scores was found between both groups. This agreed with the findings of a study by Block et al., [13] who reported that a web-based educational program for 1 month significantly reinforced nurses’ knowledge on the recommended BPM technique but not skills or attitude; improving attitudes and skills may require a mixture of live and web-based training. In contrast, a study by Rabbia et al., [12] has reported a significant improvement in BP technique after a 1-day-long face-to-face training program. Another study by Machado et al., [23] has reported a significant improvement in BP technique after a 2-h face-to-face training program. Another study has reported significant improvement in BP performance scores following the application of a bundle intervention program, which included clinical training, automated devices, and systems change support [22]. This may be because accurate BPM is affected by many barriers [35] and organization awareness and designing interventions to overcome these barriers might be needed to alter staff behaviors and attitudes [36].

In this study, before the intervention, the mean diastolic and systolic BP ROE was not within acceptable range (-2 to + 2 mmHg). This finding agrees with those in other studies, which reported unacceptable diastolic and systolic BP ROE and terminal digit bias (TDB) among nurses [11, 37]. After the intervention, the mean diastolic and systolic BP ROE significantly decreased in the intervention group with an acceptable range. This agrees with the findings of Rabbia et al., [12] who reported a significant improvement in the accuracy of systolic and diastolic BP readings after intensive training that included theoretical, audio, and video lessons and practical training. Another study has reported that after a bundled BP measurement improvement program over a 6-month period, diastolic and systolic BP with terminal digit “0” decreased from 32.1% and 33.7% to 11.1% and 11.3%, respectively [38]. In contrast, Dickson and Hajjar [11] has reported that after educational intervention, the mean systolic BP ROE and TDB were not significantly improved in the intervention group. This may be because the intervention program was brief, only lasting 2 h, and face-to-face.

### Limitations of the study

Study limitations include short follow-up period to assess knowledge, performance, and accuracy of readings outcomes. In addition, the Hawthorne effect is a potential bias in the present study. However; nurses' technique was poor at baseline. Only cardiac nurses were evaluated so generalizability of our results is unknown to other specialties or practices. Also, we don’t know whether accuracy of readings and knowledge gains were maintained during practice.

## Conclusions and recommendation

The BPM education program using WhatsApp applied in this study was effective in enhancing nurses’ knowledge and decreasing diastolic and systolic BP ROE; however, it was not effective in improving the performance scores. Improving performance may require a multimodal approach that combines both live training and distance education using VSNs tailored to the existing organizational culture and addresses barriers to adherence to BPM guidelines. In addition new evaluation methods should be adopted including video observation and use of real patients. Moreover comparing different teaching strategies is required to choose the most effective method of BPM training. Finally, Repetition of the study on a large number of similarly educated nurses and other healthcare staff is required to generalize our findings.

## Supplementary information

**Additional file 1.**

## Data Availability

The dataset supporting the conclusions of this article is included within the article (and its additional file).

## Abbreviations

BP: Blood pressure
BPM: Blood pressure measurement
VSN: Virtual social network
HTN: Hypertension
SBP: Systolic blood pressure
DBP: Diastolic blood pressure
ROE: Range of error
TDB: Terminal digit bias
